# microBEnet: Lessons Learned from Building an Interdisciplinary Scientific Community in the Online Sphere

**DOI:** 10.1371/journal.pbio.1001884

**Published:** 2014-06-17

**Authors:** Holly M. Bik, David A. Coil, Jonathan A. Eisen

**Affiliations:** 1 UC Davis Genome Center, University of California, Davis, California, United States of America; 2 Department of Evolution and Ecology, University of California, Davis, California, United States of America; 3 Department of Medical Microbiology and Immunology, University of California, Davis, California, United States of America

## Abstract

The Microbiology of the Built Environment Network (microBEnet) has served as an experiment in online community building. Here we discuss strategies used to launch a new, interdisciplinary scientific field, and their implications.

## Overview of the Microbiology of the Built Environment Network (microBEnet)

The microbiology of the Built Environment network (microBEnet: http://www.microbe.net/) was formed as an experiment in interdisciplinary community building, with an overarching goal to facilitate and nurture the development of a new research field: the microbiology of the built environment (MBE). The field of MBE research was conceived based on the reasoning that modern humans spend most of their time indoors (where they come into contact with countless microorganisms), yet we know relatively little about the microbes and microbial ecosystems that exist within buildings. The microBEnet project began in 2010 and was funded as one of the keystone initiatives in the Alfred P. Sloan Foundation's program in Microbiology of the Built Environment (http://www.sloan.org/major-program-areas/basic-research/mobe/). The first phase of microBEnet represented a close, crossdisciplinary collaboration between the Eisen laboratory at the University of California (UC), Davis (microbiology and genomics research), and Hal Levin at the Building Ecology Research Group in Santa Cruz, California (building science). In the first three years of the project, microBEnet focused on four categories of activities in order to facilitate work in the MBE field and to build a culture of openness and sharing:

1. Organizing meetings and workshops
2. Creating, curating, and sharing online resources and information
3. Enabling and educating network participants about more “open” activities in the field
4. Leveraging social media to encourage communication and collaboration

These activities have targeted audiences in three main categories:

1. Researchers funded as part of the Alfred P. Sloan Foundation's MBE program
2. Other researchers working in the MBE program, or related fields, who could gain benefits or make valuable contributions
3. “Stakeholders” such as the general public and funding agencies who make decisions that should both be influenced by and influence our understanding of the MBE

The microBEnet project has served as a new breed of forward-looking research coordination network (akin to initiatives funded through government agencies such as the National Science Foundation). Many network activities have been experimental in nature, leveraging new tools and technology to foster interdisciplinary interactions (see Box 1). In terms of goals, we strived to be nimble and dynamic during the first 3 years of the project. Online networks and the study of the MBE itself were both considered “uncharted territory”, and microBEnet was tasked with responding to community needs and suggestions. In this commentary, we discuss some of the lessons we have learned so far from microBEnet, with a view to facilitating the development of similar initiatives in other fields. We particularly focus on the role of open science activities in relation to community building efforts, public outreach and research-driven teaching projects, and the benefits and challenges of interdisciplinary interactions.

**Box 1. Activity and participation in microBEnet.**

Unless otherwise indicated, the data below are valid as of February 10, 2014.

**Website**: The microBEnet website (http://www.microbe.net/) has amassed 186,000 cumulative views during the life of the project (since launch in 2011), with steady growth in site visits over time (approximately annual doubling in page views). As an example of data from a recent month, we recorded 7,210 unique visitors in January 2014, totaling 13,000 page views. It remains difficult to quantify “participants in the network,” since this type of web data does not enable us to classify visitors according to audience (Sloan-funded researchers, other researchers in MBE fields, and public/policymaker/funding agency stakeholders).

**microBEnet Blog**: Of the 560 posts to date, 10% were guest blog posts made by people outside the microBEnet team. The majority of these guest posts were made in the last 6 months, largely through our efforts to educate people about this mode of communication. The blog comprises the home page feed of the microBEnet site (http://www.microbe.net/).

**Meetings and Workshops**: Since 2011, microBEnet has organized or sponsored over 20 meetings or workshops, with a cumulative total of over 1,000 participants attending across all events (http://microbe.net/events/category/microbenetevents/).

**Twitter**: A significant number of network participants use Twitter, many having started in the last couple years as a direct result of our efforts (https://twitter.com/phylogenomics/lists/microbenet). The microBEnet account has 266 followers (https://twitter.com/microBEnet/).

**Facebook**: The microBEnet Facebook page currently has 80 likes and 48 members in our (outdated) group (https://www.facebook.com/MicroBEnet/).

**Mendeley**: The microBEnet Mendeley collection of >800 papers currently has 110 members (http://www.mendeley.com/groups/844031/microbiology-of-the-built-environment/).

**LinkedIn**: The microbiology of the built environment group has 136 members (http://www.linkedin.com/groups/Microbiology-Built-Environment-Network-microBEnet-3317185/).

**YouTube**: The microBEnet YouTube channel has 42 subscribers and a total of 6,500 views (https://www.youtube.com/user/microBEnet/).

## Being, Building, and Teaching “Openness”

In addition to the creation of a new research field, a parallel overall goal of microBEnet is to help lead the MBE field to be a broadly open, sharing community. The importance of “openness” and educating others on how to conduct open science, has been a central theme for much of our project. This includes both creating and curating open content and building an open social media network where anyone is welcome. Although openness is not necessary and sufficient to catalyze the creation of a new research field such as the MBE, we have chosen to emphasize this concept while building microBEnet because of the potential for scientific impact, easier sustainability, and long-term utility of open resources. However, we accept that strict openness is not always necessary, and a key feature we want to encourage is sharing of any kind. As an example of the benefits of sharing, Twitter discussions have been correlated with increased downloads and early citations for manuscript preprints [1], while social media metrics have been used to determine usage patterns of scientific products (for example, journal articles that are frequently saved and read but rarely cited [2]).

The creation and curation of open content and resources serves two purposes. The first is to compile information and resources that are accessible to the entire community and hence can be broadly utilized, adopted, and built upon. For example, we are encouraging network participants to post their detailed laboratory protocols online, so that others can quickly learn the steps involved and the types of reagents or products needed (e.g., DNA extraction kits, air sampling methods). Amongst other purposes, such resources promote scientific efficiency (saving time for researchers and lowering the initial learning curve encountered when researchers need to understand a field outside their own training/discipline). The second purpose of open content is to demonstrate its long-term utility and value in regard to sustainability and longevity of resources. The open content hosted on microBEnet (“new media” content such as YouTube recordings of talks, reference collections, blog reports of meetings) is valuable because it can be rapidly distributed online and promote engagement with a broader audience (being read and shared by researchers in other fields). It also serves as a permanent resource that can be discovered via search engines (since http://www.microbe.net/ is indexed by Google) or accessed directly at any time. However, such open content may not be prioritized or produced by individual researchers. Therefore, another function of microBEnet has been to educate members of the MBE community on how and why to be more open. In addition to writing blog posts and posting presentations from our own talks, we have been instructing community members on how to blog, share their own slides, and use Twitter. Open resources and information, as well as broad distribution and exchange of information via social media, can aid in breaking down real or perceived barriers between “ingroups” and “outgroups”, leveling the playing field in terms of access, knowledge, and funding opportunities.

Each category of microBEnet task (meetings, social media, curated resources) benefits from success in the other areas. For example, meetings work best when they are publicized well, when social media is used to record meeting content and share discussions with other people, and when good communication systems (including social media) are in place for postmeeting follow-ups. As another example, curation of resources works best when information is crowd-sourced, when social media is used to share and advertise resources, and when postmeeting feedback is used to determine which resources are most useful to produce.

## Lessons Learned in Open Science

In the context of microBEnet, we have learned a number of lessons related to “openness” and “open science”. One key insight is the distinction between “free” (available at no cost) and “open” (unrestricted use and reuse) resources. Openness has advantages, but our primary goal in microBEnet has been to foster community-building in the MBE field. Thus, when choosing resources we have focused on the relevance of the available features and adoption (use and engagement) by microBEnet participants. As with everything we are doing, we are always looking for more “open” options for the various resources we curate, but in some cases proprietary platforms (for example, Twitter) are the ones that best satisfy our requirements for utility and community participation. The ubiquity of Twitter, its ease of use, and its relatively stable suite of features make it the best choice amongst social media platforms regardless of its business model. In some cases, the choice of resources has proven more complicated than anticipated. For example, a primary activity of microBEnet has been to create and continually update a reference collection of scientific literature focused around the microbiology of the built environment. When choosing what system to use, we wanted the system to have features that were “socially aware”—that is, potentially helping to build a community around the reference collection. After evaluating a few systems, we chose to focus our efforts on the Mendeley platform (see http://www.mendeley.com/groups/844031/microbiology-of-the-built-environment/), despite some dissatisfaction with its available features and user interface. The recent purchase of Mendeley by Elsevier, however, has given rise to concerns about the long-term future of Mendeley and led us to re-evaluate the available systems. We believe that an open software platform would be preferable if one was available with the right features. Thus we are now assessing features within more “open source” platforms such as Zotero (http://www.zotero.org/). Social networking tools are a key component for promoting open science, but such features might eventually come at too high a cost. Ultimately, no one can predict the long-term popularity or sustainability of any platform given the fast pace of technology, and our goal at microBEnet has always been to choose the best platform for our goals, given what is currently available. Experimentation and exploration with such tools and resources should be considered an integral part of any online community-building initiative.

Another lesson relates to social media. By allowing anyone to participate in online discussions, we have found that there is a risk of entrapment by special interests. For example, the microBEnet LinkedIn group initially hosted some productive scientific discussions, but it is now being overloaded with announcements and self-promotional material from one particular building science society. Special interests can distort the original intended use of open activities and discourage people from participating (either offending them or making them feel it is not worthwhile to contribute).

A third lesson involves variability among people. Any given participant rarely adheres to the full spectrum of “open science” activities, and it has been hard to engage every person on all fronts. For example, the Alfred P. Sloan Foundation requires submission of one microBEnet blog post per year for all built environment grantees, but this requirement has been difficult to enforce rigorously. In another example, some people may be very active on social media but have low engagement with other microBEnet resources (e.g., they decline to have their talks recorded or post their presentations to SlideShare, which consequently hinders wider dissemination of their research).

Overall, we believe there are a number of barriers to engaging network participants in open science activities: cultural status quo within scientific disciplines, time constraints, and age. Certain disciplines may be more “offline” than others; amongst microBEnet participants, building scientists have largely been unfamiliar with online tools. Social media may additionally be considered a low priority for overburdened scientists who want to concentrate on research. Studies have found age to be the most important predictor of social media use (highest among younger adults [3]), and our anecdotal evidence from microBEnet supports this trend. Students and junior scientists are more likely to be familiar with technology and view social media as an effective way to build up their professional network [4]. We also emphasize that offline discussions and networking at meetings and workshops (by nature, activities that are not “open”) are particularly critical for building professional relationship that cross disciplinary bounds. Although some network participants successfully use online tools to catalyze and maintain such in-person interactions, amongst MBE researchers this approach remains in the minority. At microBEnet, we have actively supported such offline activities and attempted to translate this type of community building into online outputs, resources, and activities (hoping that in-person interactions will eventually lead to long-term increases in open science from network participants). At meetings and workshops, microBEnet helps participants learn about available online tools and resources, share their experiences with each other, and provide feedback to microBEnet (so that we can adapt dynamically to user needs and address any gaps related to open science tools). However, microBEnet has remained the driving force behind online tools and open science activities, and this momentum has not yet gained broad traction across the wider pool of MBE researchers. Taken together, these inherent constraints consistently limit broader participation from the community.

## Reaching Out to the Public via Citizen Science: The Rise of Citizen Microbiology

Most “open science” in practice means “open to other scientists”, which is why we have worked to engage the general public in many of our efforts. From the start, one of the aims of the microBEnet project has been to organize a large citizen science project related to the microbiology of the built environment. We began by hosting a Citizen Microbiology workshop at UC Davis in January 2012, which was followed by a similar citizen science-focused symposium at the American Society for Microbiology 2013 Annual Meeting. It was out of these workshops and collaborations that the idea for Project MERCCURI (Microbial Ecology Research Combining Citizen and University Researchers on ISS) emerged.

Project MERCCURI (http://www.spacemicrobes.org/) is a collaborative citizen science project that leverages public interest in space, sports, and science to raise the profile of the microbiology of the built environment. This project has several facets. First and foremost, we are sponsoring sample collection at sporting events, collecting thousands of swabs from cellphones and shoes of the general public, as well as collecting swab samples for culturing microbes from building surfaces. After sample processing and sequencing, the resulting microbial diversity data will be publically available in an interactive, online format. A subset of microbes we collected were cultured at UC Davis and sent to the International Space Station (ISS) for a zero-gravity growth competition (dubbed the “microbial playoffs”). Via the web, the public will be able to track microbes cultured from their favorite sporting arena and see how they fare in space versus Earth. Finally, astronauts aboard the ISS will swab surfaces within their unique built environment, enabling us to sequence and analyze the “Space Station Microbiome.”

Project MERCCURI provides an example of the intersection between citizen science, openness, and social media. By promoting this project through both online and traditional media outlets, we aim to raise public awareness of microbes in the built environment. Ultimately, we hope to dispel common misconceptions (not all microbes are “bad bacteria”) and provide diverse opportunities for the public to collect and interact with scientific data.

## Research-Driven Teaching Has Many Benefits

Undergraduate research projects represent another recent focus of the microBEnet project. At UC Davis, several recent projects have enabled us to explore the microbiology of the built environment, fill gaps in the science, and train the next generation of researchers. By conducting these projects in an open manner (using blog posts, social media, and data sharing), and by constructing protocols and workflows for others, we hope to allow these gains to be widely disseminated. Here we describe one example of an undergraduate project and how it has accomplished these goals.

For the 2011/2012 academic year we conducted a project called “Sequencing Microbial Genomes from the Built Environment” (http://www.microbe.net/undergraduate-research-built-environment-genomes/). In this project, seven undergraduate students each shepherded a microbe through a complete workflow, starting with swab sampling of building surfaces, followed by culturing and isolating microbes, preparing genomic libraries for sequencing, assembling/annotating a genome, and finally publishing a genome announcement to coincide with the deposition of the microbial genome sequence in GenBank (all publications are accessible at our project website, above). The sequencing of these reference genomes provided a direct scientific benefit to the community, helping to expand the breadth of public databases. We are aiming to make the details of the workflow from start to finish openly available (manuscript currently in preparation), so that other interested scientists and educators can use them as a resource. Subsequent undergraduate projects are also underway, including research investigating microbial succession and biogeography in aquariums (http://www.microbe.net/undergraduate-research-aquarium-biogeography-and-succession/).

## The Benefits and Challenges of Interdisciplinary Interactions

The microBEnet project has acted as a facilitator between MBE-relevant fields such microbial ecology, bioinformatics, building science, engineering, and architecture, forging an interdisciplinary community. Perhaps the single most important influence of microBEnet has been to make microbiologists realize the complexities of building science, and to take these complexities into account when designing experiments and collecting data. Consequently, by exposing building scientists to ongoing work in microbial ecology (work they would likely have never seen), awareness of the biological considerations and uncertainties involved in the study of the built environment has been raised. This has prompted building scientists to work more closely with microbiologists in order to develop a more cohesive and comprehensive understanding of buildings. The scientific consequences of such interdisciplinary interactions are evident when we look at the evolution of metadata collection which has occurred during the course of the Alfred P. Sloan MBE program: early studies recorded a minimum number of environmental parameters (including perhaps temperature, pH, and location, e.g., [5]), while new projects routinely collect 15–25 different types of metadata (for example, new community requirements for “minimum” metadata standards [6]).

Because interactions between academics continue to be restricted largely to colleagues within their own subdisciplines, building an interdisciplinary scientific community has not been without challenges. microBEnet has experimented with a variety of online tools to determine how to best engage a diverse group of resources. Some were deemed failures (CiteULike—http://www.citeulike.org/was not popular for reference management) while others were more easily adopted (many microbiologists were keen to sign up for Twitter accounts). Ultimately, encouraging new interactions required curation of different types of resources to cater to researchers' personal preferences and the disciplinary status quo.

Using our experiences from the first phase of the microBEnet project, we are moving forward and now shifting our priorities towards a second phase of network building. The next steps for microBEnet will take a more focused, targeted approach to every one of our activities: for example, sponsoring smaller, focused meetings rather than general meeting sessions, and actively recruiting Alfred P. Sloan grantees to contribute to scientific products and online resources. Our goal is to target people more specifically and show network participants how they can contribute to the MBE community.

Finally, a certain amount of “lag time” is inherent when new disciplines are formed. This initial lag of traditional scientific outputs can delay scientific messages from reaching funders and regulators. In our case, true interdisciplinary publications focused on “microbiology of the built environment” are just now starting to come to fruition [5]–[7]; most studies still emphasize either microbiology or building science. Thus, while microBEnet has fostered development of a new interdisciplinary field in what we believe to be a constructive way, at present it is not clear that our message has reached funders and regulators in any substantial manner. Nonetheless, we are optimistic that the novel and interdisciplinary scientific approaches to the study of the built environment will ultimately speak for themselves.

## Abbreviations

ISS: International Space Station
MBE: microbiology of the built environment
MERCCURI: Microbial Ecology Research Combining Citizen and University Researchers on ISS
microBEnet: microbiology of the Built Environment network
UC: University of California
